# ﻿Three new species of *Lichomolgella* Sars G.O., 1918 (Copepoda, Cyclopoida, Sabelliphilidae) associated with Bryozoa in Korea

**DOI:** 10.3897/zookeys.1244.155561

**Published:** 2025-07-10

**Authors:** Jimin Lee, Il-Hoi Kim

**Affiliations:** 1 Climate Response & Ecosystem Research Department, Korea Institute of Ocean Science & Technology, Busan 49111, Republic of Korea Climate Response & Ecosystem Research Department, Korea Institute of Ocean Science & Technology Busan Republic of Korea; 2 Korea Institute of Coastal Ecology, Bucheon 14449, Republic of Korea Korea Institute of Coastal Ecology Bucheon Republic of Korea

**Keywords:** Copepod associates, coral-like bryozoan hosts

## Abstract

Three new species of *Lichomolgella* Sars G.O., 1918 are described as associates of bryozoan hosts from Korea. In all three species, the endopod of leg 1 is two-segmented, unlike the two previously known species of this genus. As additional diagnostic features, *L.exigua***sp. nov.** has a two-segmented endopod in legs 2 and 3, *L.collata***sp. nov.** has one claw plus two setae on the third segment of the antenna and an inner coxal seta on legs 2 and 3, and *L.nudicoxa***sp. nov.** lacks an inner coxal seta on all swimming legs. The genus *Lichomolgella* is transferred from the family Lichomolgidae to Sabelliphilidae and can be characterized by several diagnostic features, such as a claw in addition to setae on the second endopodal segment of the antenna, a single-segmented endopod of leg 4 bearing two distal spines, three spines plus five setae on the third exopodal segment of legs 3 and 4, a claw-like proximal scale on the mandibular gnathobase, and a vestigial distal lash of maxilla.

## ﻿Introduction

Poecilostomatoid cyclopoid copepods generally live in association with other invertebrates. The genus *Lichomolgella* Sars G.O., 1918 has been classified in the family Lichomolgidae (Humes and Stock 1973; Humes and Boxshall 1996; Boxshall and Halsey 2004) and currently consists of two known species, *L.pusilla* Sars G.O., 1918 and *L.isseli* Gallingani, 1952. *Lichomolgellapusilla*, the type species of the genus, was originally described based on a single female collected at Skudeneshavn on the southwestern coast of Norway (Sars 1918). Sars (1921) collected another female from the same locality and provided additional illustrations. In both cases, the host was unknown. Gallingani (1952) described *L.isseli*, the second species, from specimens found on *Posidonia* seagrass in Paraggi, Italy. Both species were incompletely described (Humes and Stock 1973; Humes and Boxshall 1996) and require further study.

Copepods associated with bryozoans have rarely been reported. During a general survey of intertidal invertebrates along the west and south coasts of Korea, tiny copepods were found in association with coral-like fouling bryozoans. These copepods have been revealed to be three new species of *Lichomolgella*, which are described herein.

### ﻿Materials and methods

At the type locality, several colonies of the bryozoan hosts *Celleporinaporosissima* Harmer, 1957 and another *Celleporina* species were collected from a tidal pool and placed in a plastic bottle containing 95% ethanol. Later in the laboratory the bottle was shaken and suspended material was filtered through a net. Copepods were sorted out from the filtrates. For microscopic observation selected copepod specimens were immersed in lactic acid for approximately 10 minutes and then dissected. Dissected appendages were observed using the reverse slide method of Humes and Gooding (1964). All drawings were made using a microscope equipped with a drawing apparatus and a magnification changer. Descriptions of species are based on dissected paratypes. The lengths of the appendage segments were measured as the average of the longest and shortest margins. Morphological terminology follows Huys and Boxshall (1991) for caudal setae and Humes and Boxshall (1996) for mouthparts. The type specimens have been deposited in two institutes of Korea: the Marine Biodiversity Institute of Korea (**MABIK**), Seocheon and the Honam Marine Biodiversity Institute of Korea (**HNIBR**), Mokpo.

## ﻿Results

**Order Cyclopoida Burmeister, 1834**

**Family Sabelliphilidae Gurney, 1927**

**Genus *Lichomolgella* Sars G.O., 1918**

### ﻿ Lichomolgella exigua sp. nov.

Taxon classificationAnimaliaCyclopoidaSabelliphilidae

4F717DA0-9BE9-5A5E-95F0-B491B52FE585

https://zoobank.org/29F6392A-1113-4DBD-849A-266D719275A8

Figs 1 , 2 , 3

#### Type locality.

A tidal pool at northeastern tip of Yeongjong Island (37°29'27"N, 126°35'00"E), Incheon, Korea.

#### Type material.

• ***Holotype*** (intact ♀; MABIK CR00258577) and ***paratypes*** (intact 40 ♀♀, 7 ♂♂; MABIK CR00258578) from washings of several colonies of the bryozoan *Celleporinaporosissima* Harmer, 1957, 19 April 2022, collected by I.-H. Kim. Type material has been deposited in the Marine Biodiversity Institute of Korea (MABIK), Seocheon, Korea. Dissected and figured specimens are kept in the collection of I.-H. Kim.

#### Etymology.

The specific name *exigua* is derived from the Latin *exigu* (small), referring to the small body size of the new species.

#### Description.

**Figure 1.**
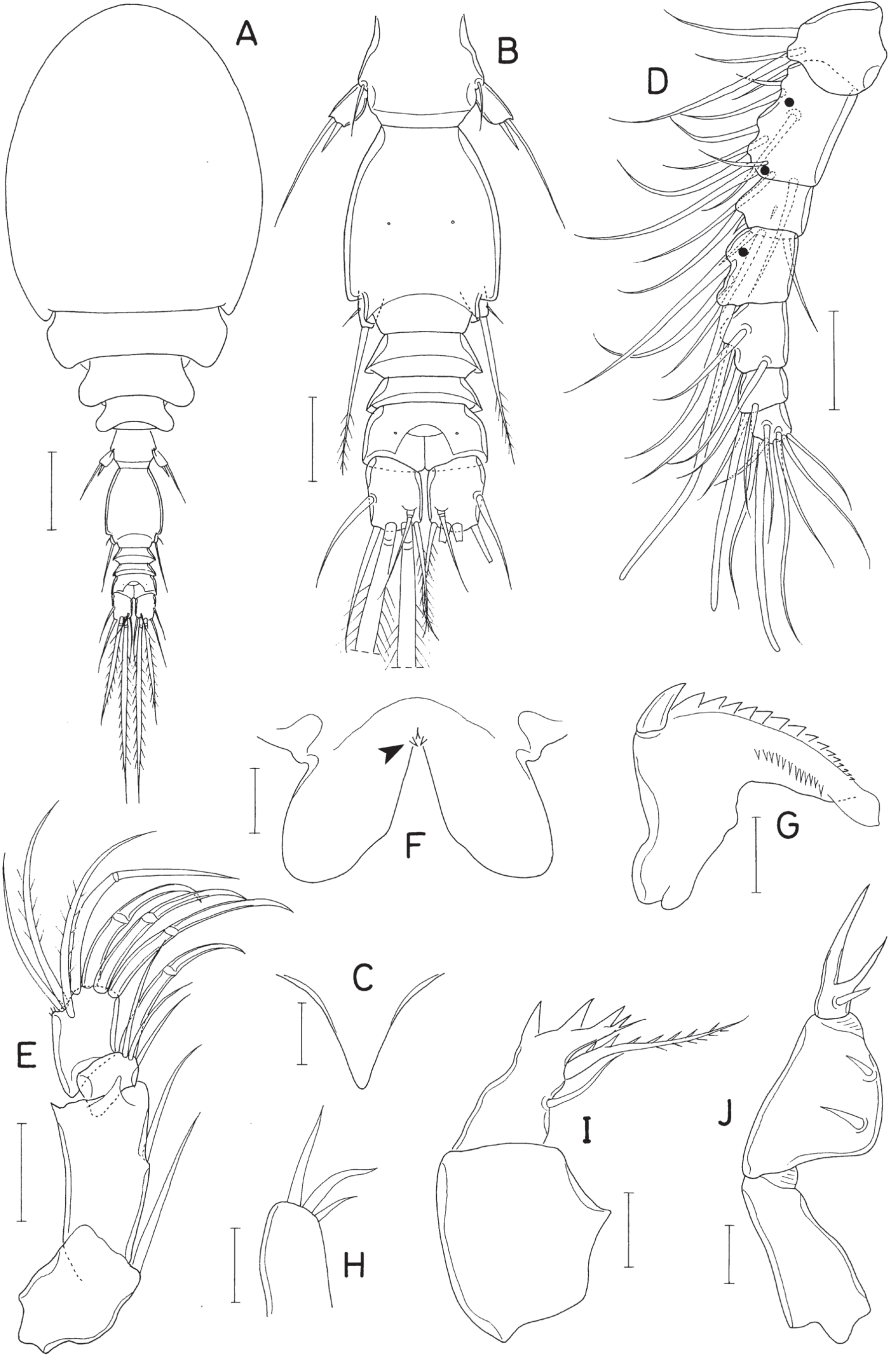
*Lichomolgellaexigua* sp. nov., female. **A.** Habitus, dorsal; **B.** Urosome, dorsal; **C.** Rostrum; **D.** Antennule; **E.** Antenna; **F.** Labrum; **G.** Mandible; **H.** Maxillule; **I.** Maxilla; **J.** Maxilliped. Scale bars: 0.05 mm (**A**); 0.02 mm (**B, D, E**); 0.01 mm (**C, F–J**).

**Figure 2.**
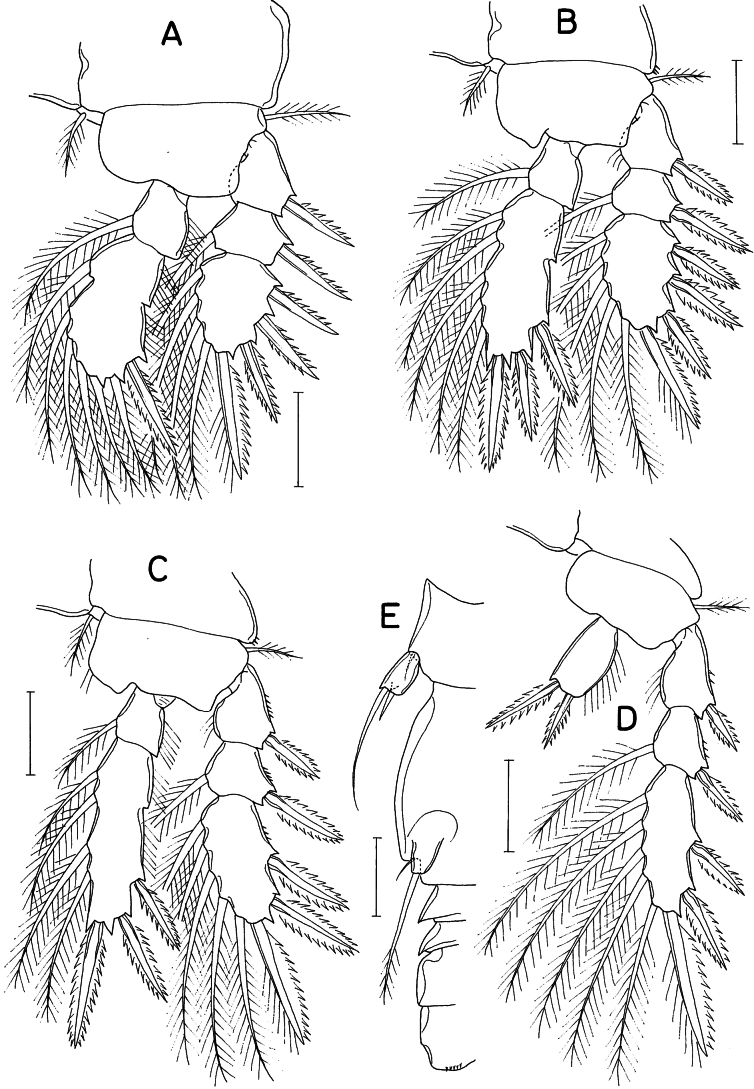
*Lichomolgellaexigua* sp. nov., female. **A.** Leg 1; **B.** Leg 2; **C.** Leg 3; **D.** Leg 4; **E.** Right side of urosome, showing leg 5 and genital aperture, ventral. Scale bars: 0.02 mm (**A–E**).

**Female.** Body (Fig. 1A) small, cyclopiform. Body length 405 μm in figured specimen, 393–430 μm in other 10 measured specimens. Prosome occupying 70% of body length. Cephalothorax 197 × 167 μm, ovoid, dorsoventrally deep, without dorsal suture line defining cephalosome and first pedigerous somite. Second to fourth pedigerous somites rapidly narrowing from anterior to posterior ones. Urosome (Fig. 1B) 5-segmented. Fifth pedigerous somite 30 μm wide. Genital double-somite distinctly longer than wide (50 × 38 μm), abruptly narrowing along posterior 15% region; genital apertures positioned ventrolaterally (Figs 1B, 2E), with large process bearing 2 very unequal setae. Three abdominal somites 10 × 28, 7 × 27, and 15 × 30 μm, respectively. Caudal ramus broad, 1.14 times longer than wide (16 × 14 μm), with 6 setae; two median terminal setae (setae IV and V) broadly flattened, tape-like; outer seta (seta II) positioned slightly distal to middle of outer margin.

Rostrum (Fig. 1C) small, strongly tapering. Antennule (Fig. 1D) short, 91 μm long, less than half length of cephalothorax, 7-segmented; armature formula 3, 11, 6, 3, 4+aesthetasc, 2+aesthetasc, and 7+aesthetasc; all setae naked. Antenna (Fig. 1E) stout, 4-segmented; armature formula 1, 1, 3+claw, and 3+4 claws; second segment (first endopodal segment) 1.74 times longer than wide (33 × 19 μm), with pointed outer distal corner; third segment (second endopodal segment) small, armed with 1 claw and 3 setae; fourth segment 1.23 times longer than wide (16 × 13 μm), characteristically attached to slightly outer region of third segment, outermost one of 4 distal claws slender, setiform.

Labrum (Fig. 1F) with rather elongated, divergent posterior lobes; each lobe bearing small denticle (indicated by an arrowhead) at proximal region of inner margin. Mandible (Fig. 1G) with short inner margin, lacking inner notch; gnathobase with 1 large claw-like scale at outer proximal corner followed by denticulate convex margin, 1 row of more than 10 small spinules along midline of one surface, and smooth concave margin; distal region of gnathobase blunt, lamellated. Maxillule (Fig. 1H) with 3 unequal, naked setae apically. Maxilla (Fig. 1I) 2-segmented; proximal segment (syncoxa) unarmed; distal segment (basis) distally with 4 teeth, distalmost one much smaller than proximal ones, and terminating in small, setule-like lash; inner seta (seta I) large, proximally broadened, with about 7 spinules along distal margin and several minute spinules along distal half of inner margin; seta II naked, slender; seta III absent. Maxilliped (Fig. 1J) 3-segmented; first segment narrow, unarmed; second segment as long as first, strongly inflated, with protruded inner margin bearing 2 naked setae, proximal seta larger than distal; third segment small, with 2 unequal, elongated spiniform processes and 1 proximal seta.

Legs 1–3 (Fig. 2A–C) each with 3-segmented exopod and 2-segmented endopod. Leg 4 (Fig. 2D) with 3-segmented exopod and 1-segmented endopod. Spines on rami large, distinctly serrate. Third exopodal segment of legs 3 and 4 armed with 3 spines and 5 setae (formula II, I, 5). Leg 4 lacking inner coxal seta; endopodal segment 1.8 times longer than wide (18 × 10 μm), with denticle-like process at inner distal corner; 2 distal spines 13 (outer) and 19 μm (inner). Armature formula for legs 1–4 as follows:

**Table:** 

	Coxa	Basis	Exopod	Endopod
Leg 1	0-1	1-0	I-0; I-1; III, I, 4	0-1; I, 2, 4
Leg 2	0-1	1-0	I-0; I-1; III, I, 5	0-1; I, II, 5
Leg 3	0-1	1-0	I-0; I-1; II, I, 5	0-1, I, II, 4
Leg 4	0-0	1-0	I-0; I-1; II, I, 5	0, II, 0

Leg 5 (Fig. 2E) consisting of 1 small dorsolateral seta on fifth pedigerous somite and small exopod; exopodal segment 11 × 6 μm, with 1 small denticle-like processes distally and armed with 2 distal setae; outer seta 31 μm long, inner seta short, spiniform, 8 μm long. Leg 6 (Fig. 2E) represented by digitiform process in genital aperture, bearing 1 small outer setule and 1 large distal seta of 33 μm long, latter seta extending to posterior margin of anal somite, pinnate along its distal third.

**Figure 3.**
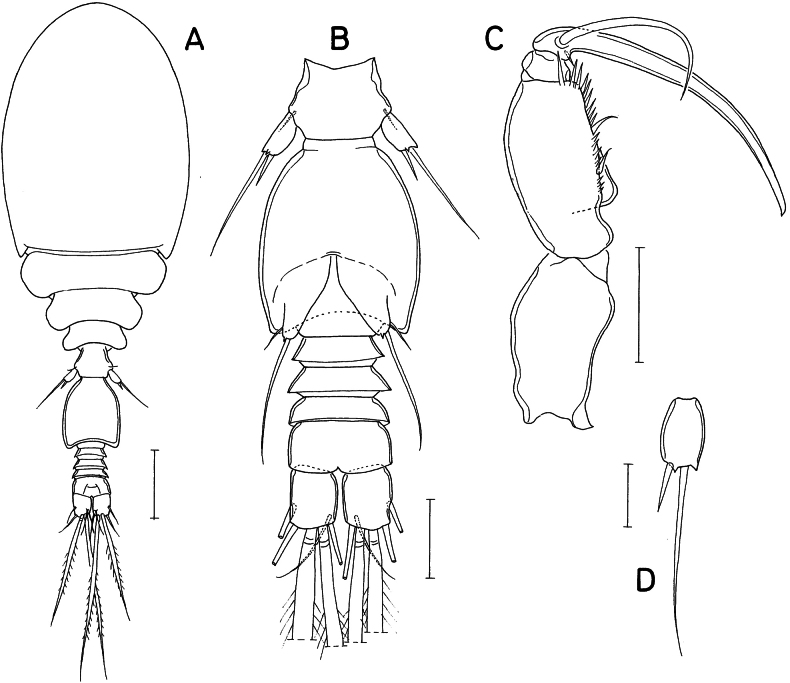
*Lichomolgellaexigua* sp. nov., male. **A.** Habitus, dorsal; **B.** Urosome, ventral; **C.** Maxilliped; **D.** Exopod of leg 5. Scale bars: 0.05 mm (**A**); 0.02 mm (**B, C**); 0.01 mm (**D**).

**Male.** Body (Fig. 3A) similar to that of female but narrower. Body length 376 μm. Cephalothorax 185 × 138 μm. Urosome (Fig. 3B) 6-segmented. Genital somite 1.2 times longer than wide (51 × 42 μm), with slightly convex lateral margins and slightly concave dorsodistal margin. Caudal ramus 1.25 times longer than wide (15 × 12 μm), armed as in female.

Rostrum as in female. Antennule with 3 additional aesthetascs, 2 on second segment and 1 on fourth segment, as indicated by dark circles in Fig. 1D. Antenna as in female, lacking additional ornamentation.

Labrum, mandible, maxillule, and maxilla as in female. Maxilliped (Fig. 3C) consisting of 3 segments and terminal claw; first segment unarmed; second segment (basis) with blunt protrusion at proximal region, 2 equally small, naked setae and row of spinules along inner margin; third segment small and unarmed; terminal claw elongate, arched, proximally bearing 1 large, naked seta and on opposite side 1 small setule.

Legs 1–4 as in female. Leg 5 (Fig. 3B) also as in female; exopodal segment (Fig. 3D) 11 × 7 μm; lengths of 2 distal setae 8 (inner) and 30 μm (outer). Leg 6 (Fig. 3B) represented by 1 large seta, 1 setule and 1 small denticle on genital operculum.

#### Remarks.

While describing *Lichomolgellaisseli*, Gallingani (1952) stated that no noteworthy differences were found in appendages between this species and the type species of the genus, *L.pusilla*, except for the antennule and antenna. According to her, *L.isseli* differed from *L.pusilla* in proportional lengths of segments of the antennule and the long second segment of the antenna. However, she did not provide specific measurements for these appendage segments. In addition, the antennule illustrated in that paper does not exhibit any taxonomic significance. However, in the illustration of the antenna, the second segment (first endopodal segment) is elongated, and Humes and Stock (1973) measured this segment as being 3.5 times longer than wide, a characteristic that allowed them to differentiate *L.isseli* from *L.pusilla*.

*Lichomolgellaexigua* sp. nov. can be clearly distinguished from the two existing species, as the endopods of legs 1–3 of the new species are all two-segmented, in contrast to the three-segmented condition in *L.pusilla* and *L.isseli*. In the forms of the antenna and genital double-somite, *L.exigua* sp. nov. is more similar to *L.pusilla* than to *L.isseli*, but still exhibits several differences from the former: (1) the fourth segment of the antenna is armed with four claws and three setae (vs one claw and five setae in *L.pusilla*); (2) the mandible is bluntly terminated (vs distally attenuated in *L.pusilla*); and (3) the third segment of the female maxilliped bears two spiniform processes and one seta (vs one spiniform process and one seta in *L.pusilla*). These and other differences between species are summarized in Table 1.

**Table 1.** Comparison of five species of *Lichomolgella* (symbols: L/W, length to width ratio; =, approximately; —, missing data).

Characters	*L.pusilla*	*L.isseli*	*L.exigua* sp. nov.	*L.collata* sp. nov.	*L.nudicoxa* sp. nov.
♀ caudal ramus, L/W	= 1.2	= 1.5	1.14	1.63	1.07
1^st^ endopodal segment of antenna, L/W	= 2	= 3.5	1.74	2.45	1.50
Armature elements on 2^nd^ endopodal segment of antenna	4	—	4	3	4
Teeth on distal margin of basis of maxilla	3	—	4	4	6 or 7
Legs bearing inner coxal seta	Leg 1	Leg 1	Legs 1–3	Legs 2 & 3	None
Endopodal segments of legs 1–3	3, 3, 3	3, 3, 3	2, 2, 2	2, 3, 3	2, 2, 2
♀ leg 5 exopod, armature	2 setae	2 setae	2 setae	Spine+seta	Spine+seta

### ﻿ Lichomolgella collata sp. nov.

Taxon classificationAnimaliaCyclopoidaSabelliphilidae

CDD460A4-15DB-5F10-A611-0781B6506820

https://zoobank.org/1F7DA53B-6DAC-48BE-BCE5-F500A42904FA

Figs 4 , 5

#### Type locality.

A tidal pool at northeastern tip of Yeongjong Island (37°29'27"N, 126°35'00"E), Incheon, Korea.

#### Type material.

• ***Holotype*** (♀, left maxilliped, left antenna, left leg 1 and left leg 2 are partially damaged; MABIK CR00258576) from washings of colonies of the bryozoan *Celleporina* sp., 11 September 2022, collected by I.-H. Kim. • ***Paratype*** (♀, dissected and figured) from the same host at type locality, 19 April 2022, collected by I.-H. Kim. Holotype has been deposited in the Marine Biodiversity Institute of Korea (MABIK), Seocheon, Korea. Dissected paratype is kept in the collection of I.-H. Kim.

#### Etymology.

The specific name *collat* (Latin, meaning “brought together”) alludes to collection of the new species together with *L.exigua* sp. nov.

#### Description.

**Figure 4.**
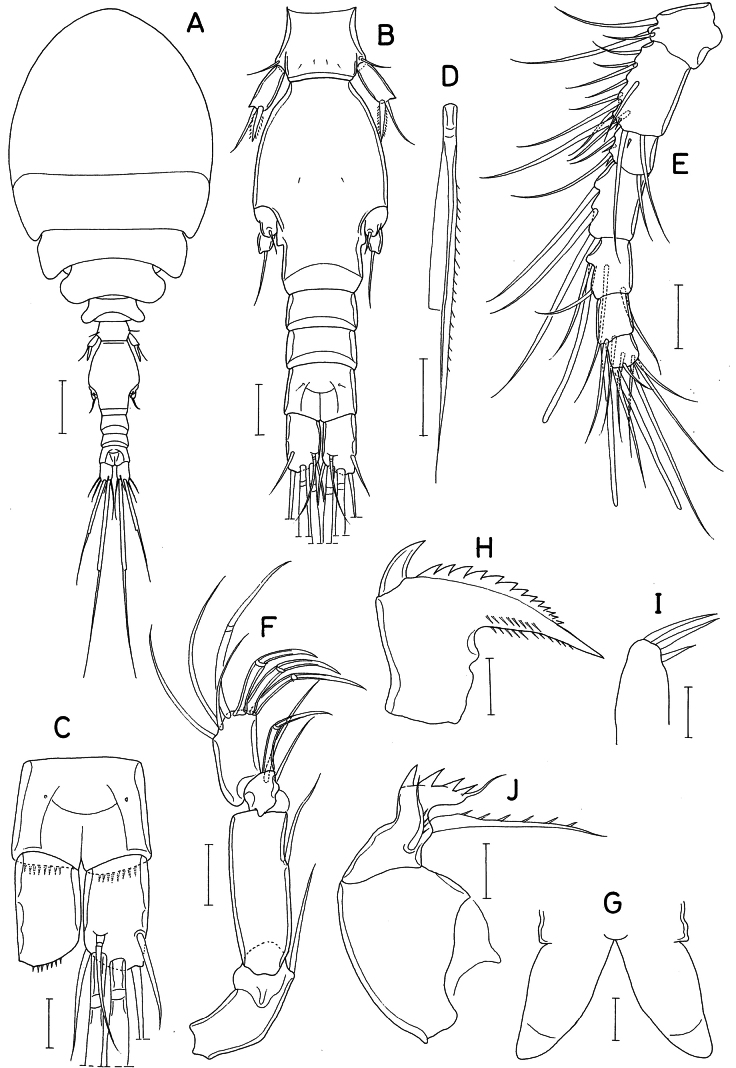
*Lichomolgellacollata* sp. nov., female. **A.** Habitus, dorsal; **B.** Urosome, dorsal; **C.** Anal somite and caudal rami, dorsal; **D.** Seta IV of caudal ramus; **E.** Antennule; **F.** Antenna; **G.** Labrum; **H.** Mandible; **I.** Maxillule; **J.** Maxilla. Scale bars: 0.05 mm (**A**); 0.02 mm (**B, D–F**); 0.01 mm (**C, G–J**).

**Female.** Body (Fig. 4A) cyclopiform. Body length of dissected paratype 450 μm. Prosome rhomboidal, 317 × 195 μm. Cephalothorax 210 μm long, longer than wide, with faint dorsal suture line between cephalosome and first pedigerous somite. Lateral corners of all prosomal somites rounded. Urosome (Fig. 4B) 5-segmented. Fifth pedigerous somite 36 μm wide. Genital double-somite 1.55 times longer than wide (82 × 53 μm), consisting of broader anterior 4/5 and narrower posterior 1/5; genital apertures positioned laterally at 70% region of double-somite. Three abdominal somites 15 × 28, 15 × 28, and 20 × 28 μm, respectively. Anal somite (Fig. 4C) with row of fine spinules along posteroventral margin. Caudal ramus (Fig. 4C) 1.63 times longer than wide (22 × 13.5 μm), with 6 setae, ornamented with several fine spinules along ventrodistal margin; setae IV (Fig. 4D) and V broadened, tape-like, feebly pinnate; other setae naked; outer seta (seta II) positioned at 75% region of outer margin of ramus.

**Figure 5.**
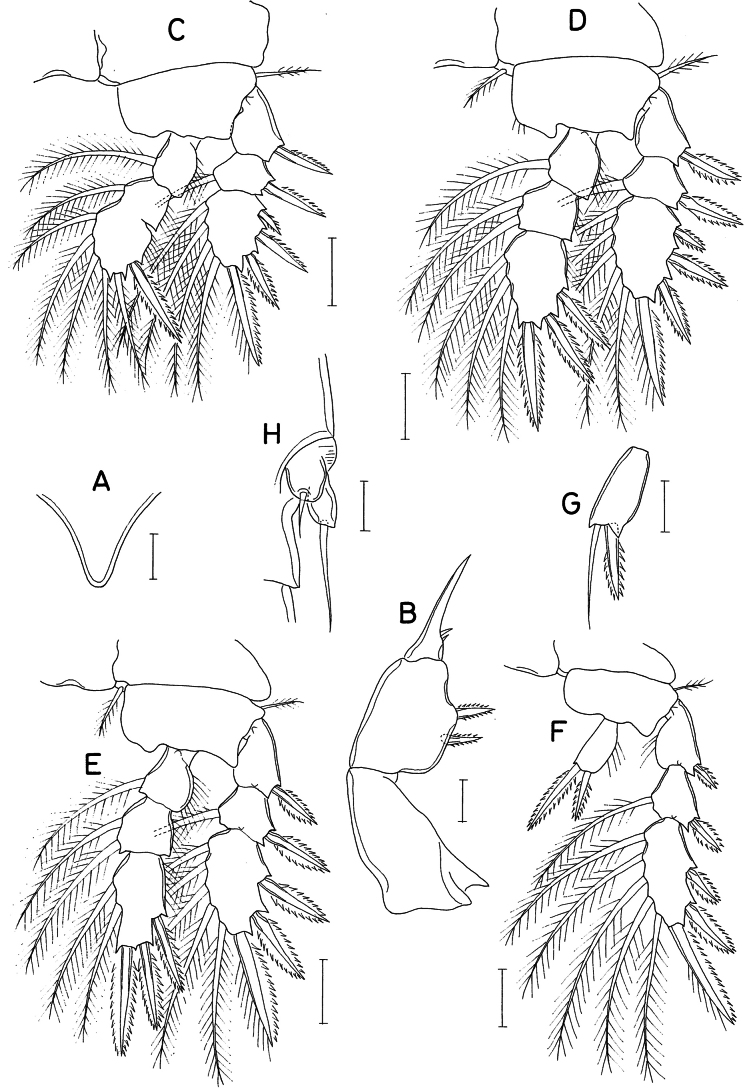
*Lichomolgellacollata* sp. nov., female. **A.** Rostrum; **B.** Maxilliped; **C.** Leg 1; **D.** Leg 2; **E.** Leg 3; **F.** Leg 4; **G.** Exopod of leg 5; **H.** Genital aperture. Scale bars: 0.01 mm (**A, B, G, H**); 0.02 mm (**C–F**).

Rostrum (Fig. 5A) strongly tapering, with blunt distal apex. Antennule (Fig. 4E) short, 119 μm long, 7-segmented; armature formula 3, 13, 6, 3, 4+aesthetasc, 2+aesthetasc, and 7+aesthetasc; all setae naked. Antenna (Fig. 4F) 4-segmented; armature formula 1, 1, 2+claw, and 3+4 claws; second segment (first endopodal segment) 2.45 times longer than wide (49 × 20 μm); fourth segment 1.67 times longer than wide (25 × 15 μm), inserted to outer side of distal margin of third segment; outermost one of 4 distal claws slender, setiform.

Labrum (Fig. 4G) with elongated, divergent posterior lobes; each lobe tapering, twice as long as wide, with transparent distal part. Mandible (Fig. 4H) with short inner margin, lacking inner notch; gnathobase with 1 large claw-like scale at outer proximal corner followed by denticulate outer margin, 2 rows of fine spinules along inner margin; distal part of gnathobase attenuated. Maxillule (Fig. 4I) with 3 naked setae at distal region. Maxilla (Fig. 4J) 2-segmented; proximal segment (syncoxa) unarmed; distal segment (basis) with 4 teeth on distal margin, small, setule-like distal lash and 2 setae; inner seta (seta I) large, proximally expanded, with about 6 spinules along distal margin; anterior seta (seta II) slender, with 3 or 4 fine spinules on inner margin; seta III absent. Maxilliped (Fig. 5B) 3-segmented; first segment longest, but unarmed; second segment with protruded inner margin, armed with 2 equal, serrated spines; third segment terminated in elongated, spiniform process, with 2 small setae proximally.

Leg 1 (Fig. 5C) with 3-segmented exopod and 2-segmented endopod; coxa lacking inner seta; first outer spine on third exopodal segment distinctly smaller than nearby spines. Legs 2 and 3 (Fig. 5D, E) with 3-segmented rami. First spine on third exopodal segment of leg 2 small, as in leg 1. Leg 3 with third exopodal segment armed with 3 spines and 5 setae (formula II, I, 5). Leg 4 (Fig. 5F) with 3-segmented exopod and 1-segmented endopod; coxa lacking inner seta; endopodal segment twice longer than wide (18 × 9 μm), armed with 2 spines distally; lengths of these spines 16 (outer) and 26 μm (inner), Armature formula for legs 1–4 as follows:

**Table:** 

	**Coxa**	**Basis**	**Exopod**	**Endopod**
Leg 1	0-0	1-0	I-0; I-1; III, I, 4	0-1; I, 2, 4
Leg 2	0-1	1-0	I-0; I-1; III, I, 5	0-1; 0-2; I, II, 3
Leg 3	0-1	1-0	I-0; I-1; II, I, 5	0-1; 0-2; I, II, 2
Leg 4	0-0	1-0	I-0; I-1; II, I, 5	0, II, 0

Leg 5 (Fig. 4B) consisting of 1 dorsolateral seta on fifth pedigerous somite and exopod; exopodal segment (Fig. 5G) 17 × 8 μm, distally with 2 dentiform processes, and armed with 1 spine (15 μm long) and 1 naked seta (21 μm long). Leg 6 (Fig. 5H) represented by 2 large lobes, each bearing 1 naked seta distally.

**Male.** Unknown.

#### Remarks.

*Lichomolgellacollata* sp. nov. can be characterized by four outstanding features: the endopod is two-segmented in leg 1 but three-segmented in legs 2 and 3; the inner coxal seta is absent in leg 1; the third segment (second endopodal segment) of the antenna is armed with one claw plus two setae (presumably resulting from the secondary loss of one seta); and the exopod of leg 5 bears one spine plus one seta (rather than two setae as in other species). Table 1 compares the new species with its congeners based on these and other distinguishing features.

Although *L.collata* sp. nov. was collected together with *L.exigua* sp. nov. from the same locality, they differ in multiple respects, including the presence of a row of fine spinules on the posteroventral margin of the anal somite in the former species. In addition to the above four distinguishing features of *L.collata* sp. nov., the latter species has a larger body, a more elongated antenna, and a mandible that is distally attenuated. Furthermore, the posterior lobes of the labrum are more elongated, the maxilliped has a different armature on the second and third segments, and the first outer spine on the third exopodal segment of legs 1 and 2 is markedly reduced in size.

### ﻿ Lichomolgella nudicoxa sp. nov.

Taxon classificationAnimaliaCyclopoidaSabelliphilidae

88FE4E15-F963-55A1-A2FE-D9122C322DD2

https://zoobank.org/67371267-8C04-4CE4-9BCF-AE773AC773F6

Figs 6 , 7

#### Type locality.

Munseom islet off Seogwipo in Jeju Island (33°13'35.5"N, 126°33'45.3"E), Korea.

#### Type material.

• ***Holotype*** (♀, HNIBRIV2392), ***paratypes*** (intact 2 ♀♀, HNIBRIV2393), and 2 dissected paratypes (2 ♀♀) from washings of a coral-like bryozoan colony, 20 July 2022, collected by Drs. Taewon Cheong, Hyun-Kyeong Kim and Jong-Guk Kim. Holotype and intact paratypes have been deposited in the Honam National Institute of Biological Resources (HNIBR), Mokpo, Korea. Dissected paratypes are kept in the collection of I.-H. Kim.

#### Etymology.

The specific name *nudicoxa* is a noun derived from Latin *nud* (naked) and *coxa* (the hip), referring to the absence of the inner coxal coxa on legs 1–4.

#### Description.

**Figure 6.**
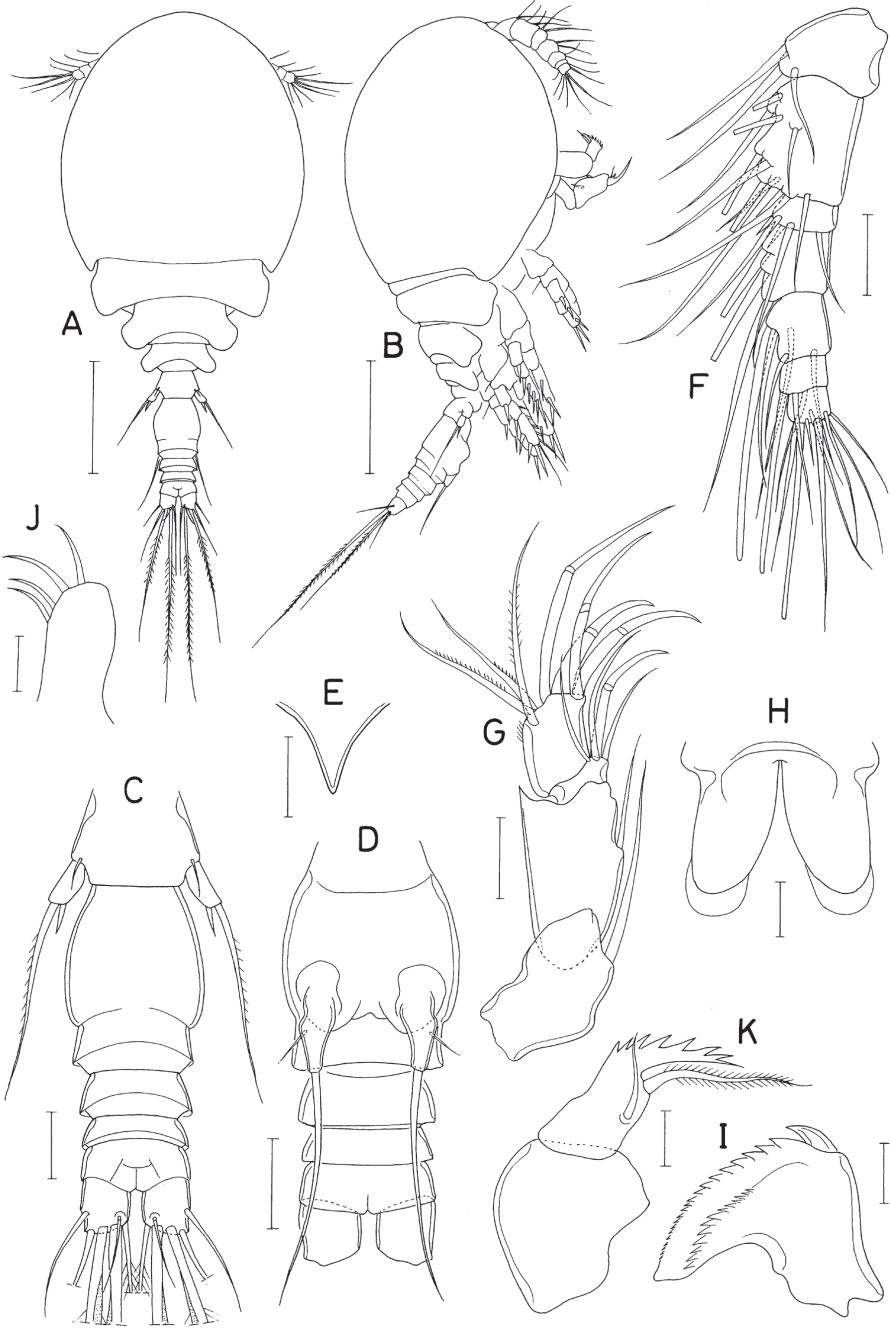
*Lichomolgellanudicoxa* sp. nov., female. **A.** Habitus, dorsal; **B.** Habitus, right; **C.** Urosome, dorsal; **D.** Genital double-somite and abdomen, ventral; **E.** Rostrum; **F.** Antennule; **G.** Antenna; **H.** Labrum; **I.** Mandible; **J.** Maxillule; **K.** Maxilla. Scale bars: 0.1 mm (**A, B**); 0.02 mm (**C–G**); 0.01 mm (**H–K**).

**Female.** Body (Fig. 6A, B) small, cyclopiform. Body length of described specimen 447 μm. Prosome rhomboidal, 314 × 220 μm. Cephalothorax 220 μm long, as long as wide, lacking dorsal suture line defining cephalosome and first pedigerous somite. Lateral corners of all prosomal somites rounded or blunt. Urosome (Fig. 6C) 5-segmented. Fifth pedigerous somite 38 μm wide. Genital double-somite 1.24 times longer than wide (52 × 41 μm), consisting of broader anterior 4/5 and narrower posterior 1/5, with partial articulation dorsally between these two parts; genital apertures positioned ventrally (Fig. 6D), represented by large digitiform process tipped with large seta (59 μm long, extending beyond distal margin of caudal rami). Three abdominal somites 15 × 33, 9 × 32, and 12 × 33 μm, respectively. Anal somite with smooth posteroventral margin (Fig. 6D). Caudal ramus 1.07 times longer than wide (16 × 15 μm), with 6 setae, unornamented; setae IV and V broadened, tape-like; setae IV–VI weakly pinnate, other setae naked.

Rostrum (Fig. 6E) strongly tapering, with pointed distal apex. Antennule (Fig. 6F) stout, 103 μm long, 7-segmented; armature formula 3, 11, 5, 3, 4+aesthetasc, 2+aesthetasc, and 7+aesthetasc; all setae naked. Antenna (Fig. 6G) 4-segmented; armature formula 1, 1, 3+claw, and 3+4 claws; second segment (first endopodal segment) 1.50 times longer than wide (36 × 24 μm), with acutely pointed outer distal corner; third segment very short; fourth segment 1.31 times longer than wide (21 × 16 μm).

**Figure 7.**
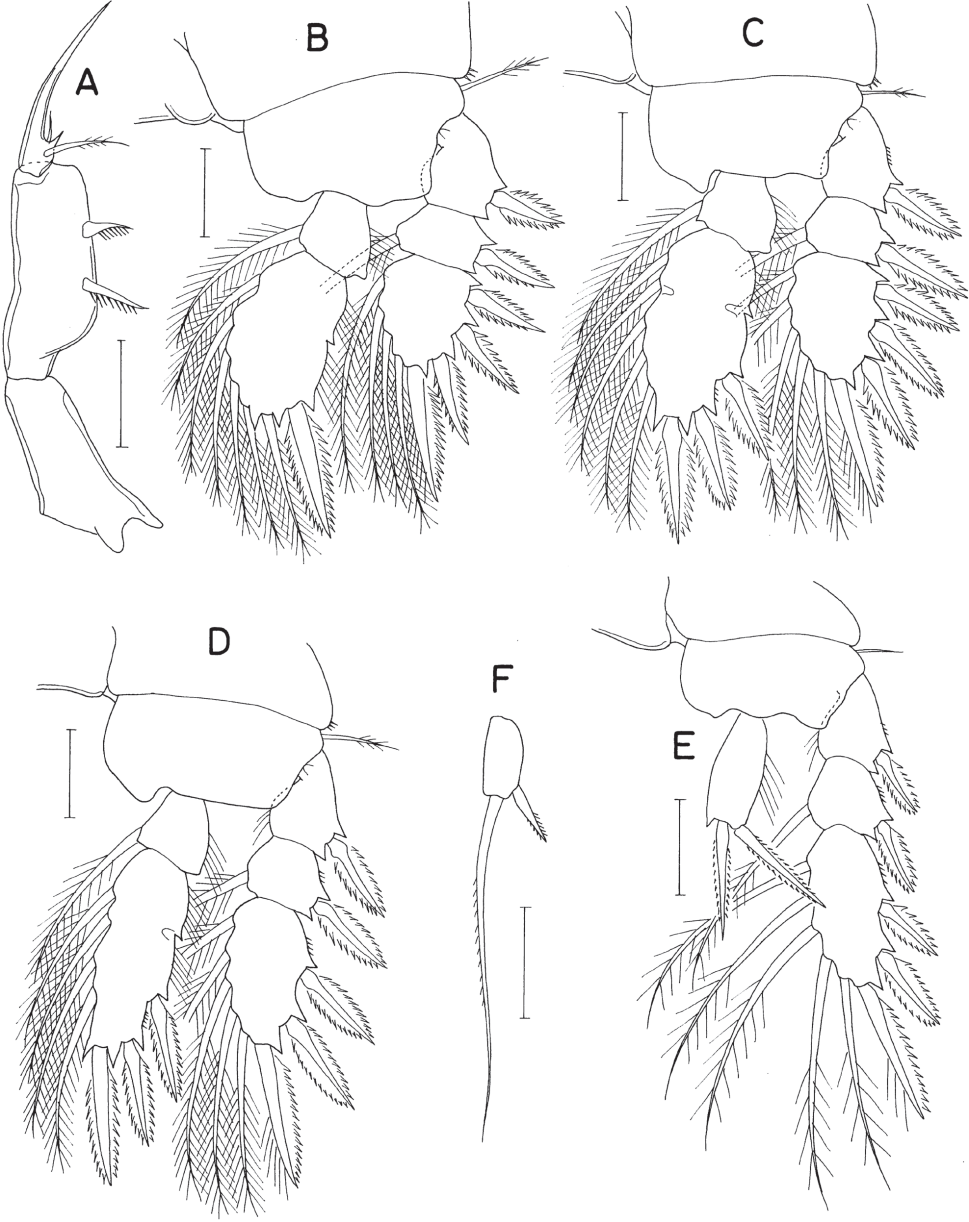
*Lichomolgellanudicoxa* sp. nov., female. **A.** Maxilliped; **B.** Leg 1; **C.** Leg 2; **D.** Leg 3; **E.** Leg 4; **F.** Exopod of leg 5. Scale bars: 0.02 mm (**A–F**).

Labrum (Fig. 6H) with elongate posterior lobes and deep median incision; each lobe much longer than wide, with broad membranous fringe on distal margin. Mandible (Fig. 6I) lacking inner notch; gnathobase short, bluntly tipped, with 1 large claw-like scale at outer proximal corner followed by denticulate outer margin, 1 row of denticles on anterior surface. Maxillule (Fig. 6J) lobate, with 4 naked setae. Maxilla (Fig. 6K) 2-segmented; proximal segment (syncoxa) unarmed; distal segment (basis) with 2 setae (setae I and II), 6 or 7 teeth on distal margin and setule-like distal lash; inner seta (seta I) large, pinnate, extending beyond distal lash; anterior seta (seta II) slender, unornamented; seta III absent. Maxilliped (Fig. 7A) 3-segmented; first segment unarmed; second segment armed with 2 unilaterally spinulose setae, 14 (proximal one) and 9 μm (distal one), respectively; third segment terminated in elongated, spiniform process, proximally with 2 unequal setae and 1 small spiniform process.

Legs 1–3 (Fig. 7B–D) with 3-segmented exopod and 2-segmented endopod. Leg 4 (Fig. 7E) with 3-segmented exopod and 1-segmented endopod. Coxa of legs 1–4 lacking inner seta. Third exopodal segment of legs 3 and 4 armed with 3 spines and 5 setae (formula II, I, 5). Endopodal segment of leg 4 twice as long as wide; distally armed with 2 spines of equal length (25 μm). Armature formulae for legs 1–4 as follows:

**Table:** 

	Coxa	Basis	Exopod	Endopod
Leg 1	0-0	1-0	I-0; I-1; III, I, 4	0-1; I, 1, 5
Leg 2	0-0	1-0	I-0; I-1; III, I, 5	0-1; I, II, 5
Leg 3	0-0	1-0	I-0; I-1; II, I, 5	0-1; I, II, 4
Leg 4	0-0	1-0	I-0; I-1; II, I, 5	0, II, 0

Leg 5 (Fig. 6C) consisting of 1 small dorsolateral seta on fifth pedigerous somite and exopod; exopodal segment (Fig. 7F) 2.0 times longer than wide (14 × 7 μm), distally armed with 1 spine (11 μm long) and 1 long seta (62 μm long). Leg 6 (Fig. 6D) probably represented by large digitiform process bearing 1 small lateral and 1 large distal seta on genital operculum.

**Male.** Unknown.

#### Remarks.

*Lichomolgellanudicoxa* sp. nov. has two-segmented endopods of legs 1–3, a segmentation pattern shared only with *L.exigua* sp. nov. However, *L.nudicoxa* sp. nov. is clearly distinguished from the latter species, as it has no inner coxal setae in legs 1–3 (Table 1). The presence of a large distal seta on the female genital operculum, which extends beyond the distal margin of the caudal rami, appears to be an outstanding feature of *L.nudicoxa* sp. nov. A similar distal seta on the female genital operculum is observed in *L.pusilla* and *L.exigua* sp. nov., but it is distinctly smaller in these two species, extending beyond the posterior margin of the first free abdominal somite in *L.pusilla* as illustrated by Sars (1918) or reaching the posterior margin of the anal somite in *L.exigua* sp. nov. as shown in Fig. 1B. The presence of six or seven teeth on the distal margin of the basis of the maxilla is another characteristic feature of *L.nudicoxa* sp. nov. In contrast, the number of the teeth on the same margin is three in *L.pusilla*, as illustrated by Sars (1921), and four in both *L.exigua* sp. nov. and *L.collata* sp. nov.

## ﻿Discussion

In the original description of the type species, *Lichomolgellapusilla*, Sars (1918) noted that this species has a single-segmented endopod in leg 4, as is usual for the genus *Pseudanthessius* Claus, 1889. However, he placed the species in a new genus, *Lichomolgella*, due to the presence of a free exopod in leg 5. *Lichomolgella* has since been incorporated into the family Lichomolgidae (Humes and Stock 1973; Humes and Boxshall 1996; Boxshall and Halsey 2004). The present discovery of three new species of *Lichomolgella* necessitates a reconsideration of the familial position of this genus. Humes and Boxshall (1996) redefined the families in the superfamily Lichomolgoidea Humes & Stock, 1972. In defining the family Sabelliphilidae, they emphasized characteristic morphological features exhibited in the antenna and mandible of genera of the family, i.e., (1) the second endopodal segment of the antenna bears four setal elements, (2) one of these elements is modified into a claw, (3) the gnathobase of the mandible is short and strongly tapering, and (4) the convex outer margin of the mandible bears a well-defined proximal scale. All three new species of *Lichomolgella* described herein exhibit these features, except for *L.collata* sp. nov., in which one of the setae on the second endopodal segment of the antenna is lost. The presence of most of these typical sabelliphilid morphological features in the three new species suggests that the genus should be placed in the family Sabelliphilidae. Additionally, *Lichomolgella* shares two other characteristics with some genera of the Sabelliphilidae, such as the type genus *Sabelliphilus* Sars M., 1862, *Acaenomolgus* Humes & Stock, 1972, *Myxomolgus* Humes & Stock, 1972, and *Nasomolgus* Sewell, 1949. These include the presence of elongated posterior lobes of the labrum and a markedly shortened distal lash of the basis (second segment) of the maxilla. These features further support the placement of *Lichomolgella* within the Sabelliphilidae.

Within the Sabelliphilidae, *Lichomolgella* is easily differentiated from most other genera of the family by two diagnostic features: the third endopodal segment of leg 3 is armed with three spines plus five setae (formula II, I, 5), and the endopod of leg 4 is single-segmented. The first diagnostic feature is shared with two genera, *Nasomolgus* and *Phoronicola* Boxshall & Humes, 1988, while the second is shared only with *Phoronicola*. However, *Lichomolgella* is distinct from the two genera, because in *Nasomolgus* the labrum has a pair of large setae, the female maxilliped bears a long, whip-like terminal segment, and the endopod of leg 4 is two-segmented, while in *Phoronicola*, the urosome is four-segmented in the female and five-segmented in the male, the endopod of leg 4 bears a single spine, and the third exopodal segment of leg 4 is armed with two spines plus five setae (formula I, I, 5, compared to II, I, 5 in *Lichomolgella*).

Members of the Sabelliphilidae are generally associated with polychaete worms, with a few exceptions. Species of *Eupolymniphilus* Humes & Boxshall, 1996 have uncertain hosts, and *Phoronicolaspinulatus* Boxshall & Humes, 1988 has been found on a phoronid worm in Hong Kong (Boxshall and Humes 1988) or on a mixture of a phoronid and a sea anemone in New Caledonia (Kim and Huys 2012). The hosts of *Lichomolgella* remained unknown (Humes and Boxshall 1996) until the present study. Thus, our discovery of three new species of *Lichomolgella* associated with bryozoan hosts is remarkable, as no previous species of Sabelliphilidae were known to be associated with bryozoans. Gallingani (1952) collected *L.isseli* from seagrass of the genus *Posidonia* König, 1805, but it is highly likely that the true host of this copepod was a bryozoan epiphytic on that seagrass.

A comparison of the five known species of *Lichomolgella* has enabled us to redefine the genus, as follows:

### Revised diagnosis of *Lichomolgella*

Body small, cyclopiform. Prosome expanded dorsally and laterally. Urosome 5-segmented in female and 6-segmented in male. Caudal ramus with 6 setae. Antennule short, 7-segmented; first segment with 3 setae; male antennule with 3 additional aesthetascs, 2 on second segment and 1 on fourth segment. Antenna 4-segmented; third segment (second endopodal segment) with 1 claw plus 2 or 3 setae; fourth segment obliquely inserted into third segment, with 7 elements including 1 to 4 claws. Labrum with elongated posterior lobes. Mandible with strongly tapering gnathobase, with 1 large claw-like scale proximally on convex margin of gnathobase. Maxillule with 3 or 4 setae. Maxilla with several teeth on distal margin of second segment (basis); distal lash rudimentary; setae I and II distinct. Female maxilliped 3-segmented; third segment with 1 or 2 spiniform processes and 1 or 2 setae. Male maxilliped 4-segmented; terminal segment as long claw. Legs 1–3 with 3-segmented exopods and 2- or 3-segmented endopods. Leg 4 with 3-segmented exopod and 1-segmented endopod; endopod bearing 2 spines distally. Third exopodal segment of legs 3 and 4 with 3 spines and 5 setae (formula II, I, 5). Exopod of leg 5 small but free, armed with 2 setae or 1 spine plus 1 seta. No sexual dimorphism in legs 1–4. Associated with Bryozoa.

## Supplementary Material

XML Treatment for Lichomolgella exigua

XML Treatment for Lichomolgella collata

XML Treatment for Lichomolgella nudicoxa

## ﻿Additional information

### Use of AI

No use of AI was reported.

### Author ORCIDs

Jimin Lee https://orcid.org/0000-0001-9004-8275

Il-Hoi Kim https://orcid.org/0000-0002-7332-0043

### Data availability

All of the data that support the findings of this study are available in the main text.
